# The impact of obesity on upper airway anatomy as assessed by magnetic resonance imaging and obstructive sleep apnea endotypic traits

**DOI:** 10.3389/fphys.2025.1648767

**Published:** 2025-10-01

**Authors:** Brandon Nokes, Aaron Schueler, Chantal Darquenne, Cristopher N. Schmickl, Brian S. Wojeck, Stacie Moore, Pamela Deyoung, Lana McGinnis, Rebecca J. Theilmann, Eli Gruenberg, Eduardo Grunvald, Breanna M. Holloway, Raichel M. Alex, Scott Sands, Peter Colvonen, Robert L. Owens, Atul Malhotra

**Affiliations:** ^1^ Sleep Medicine Section, Veterans Affairs San Diego Healthcare System, San Diego, CA, United States; ^2^ Division of Pulmonary, Critical Care, Sleep Medicine and Physiology, University of California, San Diego (UCSD), San Diego, CA, United States; ^3^ Section of Endocrinology, Yale School of Medicine, New Haven, CT, United States; ^4^ Department of Radiology, University of California, San Diego, CA, United States; ^5^ Division of General Internal Medicine, University of California, San Diego (UCSD), San Diego, CA, United States; ^6^ Department of Neurosciences, University of California San Diego, San Diego, CA, United States; ^7^ Division of Sleep and Circadian Disorders, Brigham and Women’s Hospital and Harvard Medical School, Boston, MA, United States; ^8^ VA Center of Excellence for Stress and Mental Health (CESAMH), San Diego, CA, United States

**Keywords:** osa, obesity, MRI, endotype, endotypes, upper airway

## Abstract

**Introduction:**

Obesity is an important risk factor for obstructive sleep apnea (OSA) development. Likewise, obesity management is an important component of OSA treatment. We sought to evaluate the OSA endotypes as well as upper airway anatomy, using magnetic resonance imaging (MRI) in patients referred from a bariatric surgery clinic.

**Methods:**

The SLIM-OSA trial (NCT04793334; IRB#191948) seeks to elucidate the mechanisms for why weight loss improves OSA in some but not all individuals. Participants underwent baseline research polysomnography. Six months following sleeve gastrectomy for those who underwent surgery, polysomnography was repeated. A subset of these individuals also completed upper airway magnetic resonance imaging (MRI). We evaluated relationships between upper airway anatomy and endotypic traits.

**Results:**

Of 55 individuals undergoing baseline studies, 22 completed upper airway MRI and polysomnography at baseline, with 5 individuals returning for MRI and polysomnography after sleeve gastrectomy. The study population was 86.4% female, with a mean age of 41.7 (11) years and median AHI of 11/h [IQR 2, 33]. Upper airway length was strongly associated with apnea hypopnea index (AHI), hypoxic burden, and ventilatory burden; somewhat surprisingly, tongue fat percentage was not associated with AHI.

**Conclusion:**

The relationship between obesity and OSA is complex and likely evolves through multiple mechanistic avenues. These findings may help inform future mechanistic studies aimed at understanding the heterogeneous impact of weight loss on OSA outcomes.

## Introduction

Obstructive sleep apnea (OSA) is very common and arises from multiple patient-level risk factors and pathophysiologic mechanisms (Benjafield et al., 2019; Edwards et al., 2019). One important risk factor for OSA development is obesity, which is a major driver of OSA pathogenesis in about half the patients, exerting its effects through multiple pathways as body mass index (BMI) increases (Hang et al., 2024; Yao et al., 2025). The SLIM-OSA trial (NCT04793334) seeks to evaluate how obesity influences OSA pathogenesis and how these relationships change following sleeve gastrectomy as assessed by both magnetic resonance imaging (MRI) of the upper airway as well as OSA endotypes.

Briefly, the OSA endotypes are pathophysiologic mechanisms contributing to OSA development. The endotypes are still evolving but currently include: passive airway collapsibility traits—ventilation through the passive airway at eupneic ventilatory drive (Vpassive) and at nadir ventilatory drive (Vmin); ventilation with maximal muscle recruitment (Vactive); the ventilatory drive level at which arousal occurs (respiratory arousal threshold); and ventilatory control instability (loop gain) (Sands et al., 2018).

We have previously reported on the baseline (pre-operative) characteristics of individuals seeking bariatric surgery at our institution as part of SLIM-OSA (Nokes et al., 2024). We determined that of individuals with class 2 obesity (BMI>35 to<40 kg/m^2^) and class 3 obesity (BMI>40 kg/m^2^) who developed OSA, OSA was primarily caused by a more collapsible upper airway both during passive and active conditions as measured by the OSA endotypes. This finding was in contrast to prior studies including subjects who were overweight (BMI>25 to<30 kg/m^2^) or had class 1 obesity (BMI>30 to<35 kg/m^2^), where OSA pathogenesis appears to be driven by upper airway responsiveness to flow limitation, so-called upper airway gain (UAG) in addition to a more collapsible airway (Sands et al., 2014).

We subsequently conducted a multiple mediation analysis in the retrospective San Diego Multi-Outcome OSA Endophenotype (SNOOzzzE) cohort to investigate how obesity influences the apnea-hypopnea index (AHI) through OSA endotypes (Nokes et al., 1985). This analysis suggested that the effect of obesity on OSA is modified by age and sex. In men and pre-menopausal women, the effect of BMI on AHI was primarily mediated by increased upper airway collapsibility. In contrast, in women in the menopausal and post-menopausal age range, the primary mediator was chemoreflex delay (circulation time between the lungs and chemoreceptors), which was inversely associated with BMI: a higher BMI appeared to lead to a higher cardiac output, thus shortening chemoreflex delay, which in turn leads to shorter but more frequent events (Borker et al., 2021).

With respect to imaging the upper airway to make anatomical inferences regarding obesity and OSA pathogenesis, prior work has indicated that tongue fat percentage changes with AHI severity and improves with weight loss, irrespective of whether weight loss was medical or surgical (Wang et al., 2020). Thus, we hypothesized that tongue fat percentage, as assessed by awake-state MRI, would be predictive of apnea-hypopnea index (AHI) and Vmin during non-REM (NREM) sleep (a non-invasive surrogate for critical closing pressure (PCRIT)).

## Methods

### Subjects

For this single-center trial, we enrolled participants 18–65 years of age with BMIs>35 kg/m^2^, who were nonsmokers, without major comorbidities, and who planned to undergo sleeve gastrectomy. Recruitment was from both the University of California, San Diego bariatric and sleep clinics. Exclusion criteria were any cardiovascular, pulmonary or renal disease other than well-controlled hypertension or asthma, pregnancy, current smoking, any respiratory disorder other than OSA or well controlled asthma, or contraindication to MRI.

**FIGURE 1:**
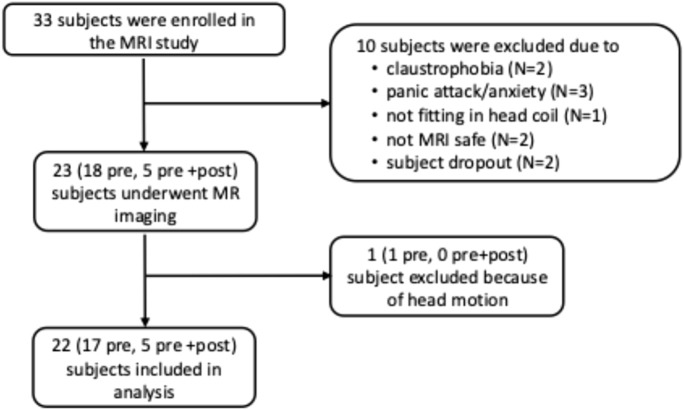
Flowchart of enrollment of participants in the MR portion of the study. Pre: MR study pre-bariatric surgery only; pre + post: MR study performed both pre- and post-bariatric surgery.

Of the 55 participants in the OSA pathophysiology study (IRB #191948), 33 were enrolled in the MRI portion. Ten of these 33 subjects did not undergo MRI due to claustrophobia (N = 2), panic attack or anxiety (N = 3), inability to fit in the head coil (N = 1), MRI incompatibility (N = 2), or dropout (N = 2). Baseline MRI was successfully performed in the remaining 23 subjects, including 18 who had imaging pre-surgery only and 5 who had both pre- and post-surgery imaging. One subject (pre-surgery only) was excluded from analysis due to excessive head motion. Therefore, MRI data from 22 subjects (17 pre-surgery only, 5 pre- and post-surgery) were included in the final analysis (Figure 1).

### Procedures

#### Research polysomnography

In-lab polysomnography was completed using Nox A1 equipment (Atlanta, GA) in the supine position. A blinded registered polysomnographic technologist scored the AHI according to American Academy of Sleep Medicine (AASM) 1a criteria (hypopneas were defined as a 30% decrease in airflow for 10 or more seconds associated with an oxygen desaturation of>3% or arousal). Signal data were stored in European Data Format (EDF) and scoring data were stored as excel (.xls) files.

#### Endotype measurements

**FIGURE 2:**
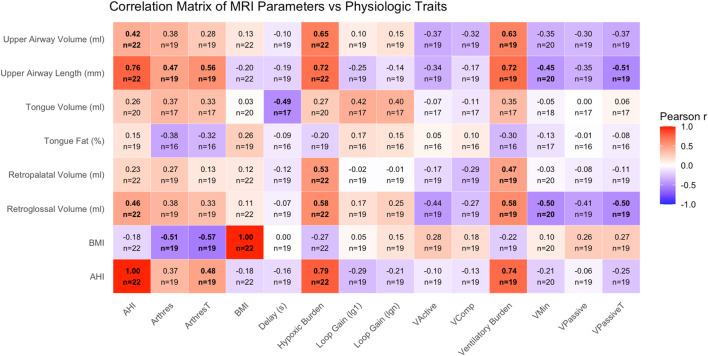
Correlation matrix displaying pairwise Pearson correlation coefficients (r) between MRI-derived upper airway traits (rows) and PSG-derived physiological OSA traits (columns). Color intensity and direction indicate strength and direction of the correlation, with values ranging from −1 to +1. MRI traits include tongue volume and fat percentage, regional airway volumes, and airway length. PSG traits include markers of OSA severity (AHI, HBtotal), ventilatory control (LG1, LGN, chemoreflex delay), arousal threshold, and anatomical endotypes (Vpassive, Vactive, Vcomp, etc.). BMI and AHI are included on both axes. Within each box, the number available comparisons is denoted. Bolded boxes indicate a p-value less than 0.05.

All studies were processed using established approaches by assessing raw polysomnography signals during NREM sleep in the supine position (Sands et al., 2018). To determine upper airway collapsibility, we quantified Vmin, which estimates the flow as a percentage of eupneic ventilation (%Veupnea) at nadir drive. Vmin correlates well with passive closing pressure (Pcrit) (r = −0.54) which is the standard to assess upper airway collapsibility (Carberry et al., 2016; Vena et al., 2022). Vpassive, was estimated as the flow at eupneic drive (i.e., lower Vmin/Vpassive denote worse collapsibility). To assess responsiveness of upper airway dilator muscles, we quantified Vactive (flow at the level of the arousal threshold), Vcomp (the change in flow from passive to active conditions (Vactive-Vpassive)). Ventilatory control instability (“loop gain”) was estimated as the magnitude of the ventilatory drive response to a prior reduction in ventilation in the time frame of 1 min (loop gain 1) and the natural frequency (loop gain n; range 0 to infinity, higher levels of loop gain denote more instability). The arousal threshold was estimated as the level of respiratory drive causing arousals (lower values reflecting easier arousability). The ventilatory response to arousal was estimated as the ventilatory overshoot (%Veupnea) in response to arousals (Edwards et al., 2013; Jordan et al., 2004), and circulatory delay was estimated as the latency between a drop in ventilation and a subsequent rise in chemical drive (the circulation time between the lung and chemoreceptors) (Terrill et al., 2015). Ventilatory burden is an event specific area under “ventilation” curve, using data from breaths within scored events that are 90% or less of eupnea (%eupnea x time/hour of sleep) (Staykov et al., 2024). This is a PUPbeta modification of the ventilatory burden put forth by Parekh et al (% breaths with <50% normalized amplitude) (Parekh et al., 2023). Hypoxic burden (HB) reflects the area under the curve of desaturation events over the course of the night (expressed in %min/h) (Azarbarzin et al., 2019). Vpassive (VpassiveT = 100 – 100*sqrt [1-Vpassive/100]) and Arousal Threshold (Arousal ThresholdT = 100 + 100*sqrt [ArTH/100–1]) were transformed for normality. A representative example of endotype measurement from polysomnography is shown in Figure 2. A supplemental table (Supplementary Table S1) of endotype measurements is also available.

MR study - After screening for MR compatibility, the subject was fitted with ear plugs and positioned in the scanner in the supine position with their head positioned with the Frankfort plane perpendicular to the scanner table. Small cushions were inserted between the coil and the head of the subject to minimize motion during imaging. MRI was performed on a 1.5T General Electric (GE) HDx EXCITE clinical MRI scanner using a neurovascular coil. MRI measurements were during awake state. The length of the upper airway was defined as the distance between the hard palate and the base of the epiglottis (Darquenne et al., 2024).

Volumetric imaging of the upper airway was performed by acquiring a T1-weighted volume using a 3D spoiled gradient-echo MR sequence with the following sequences parameters: acquisition matrix = 256 x 256 x number of slices adapted to encompass the head in the right-to-left direction, TE = 3.8 m, flip angle = 10°, TR = 8.5 m and FOV= 25.6 cm. Data were constructed to a voxel size of 1 mm^3^. This protocol is similar to that used previously (Darquenne et al., 2024).

To assess fat content as a percentage in the tongue, paired in-phase and out-of-phase MRI gradient echo (GRE) sequences were also obtained with the following parameters: flip angle = 90°, TR = 500 m and FOV= 25.6 cm. The in-phase and out-of-phase datasets were both acquired in approximately 2.5 min with a TE = 4.2 m and TE = 2.3 msec, respectively. Volumes representing fat content were created using the Dixon method: fat only volume = ½ (in-phase volume–out-of-phase volume) (Wang et al., 2020; Orr et al., 2021).

### Data analysis

MRI study - The upper airway and tongue anatomy were manually segmented for each subject’s volumetric data utilizing the 3D Visualization and Analysis AMIRA software (ThermoFisher Scientific). Tissue volume was estimated by summing the number of voxels within the corresponding region of interest where each voxel represents 0.001 mL of tissue.

Similar to procedures previously published (Wang et al., 2020; Kim et al., 2014) the “fat only” volume was scaled on a subject-by-subject basis to determine the voxel intensity range that best represents the absence of fat (0% fat) and consists entirely of fat (100%) within a voxel. The absence of fat (0% fat) was determined by calculating three times the standard deviation of the signal within a large ROI placed in outside of head “fat only” image (e.g., noise). The brightest signal of chin fat external to the tongue was selected to represent 100% fat within a voxel. Tongue fat content (%) was obtained by integrating the scaled signal intensity and dividing by the number of voxels within the tongue region of interest. The analysis of the MRI images was performed by a single individual (AS), which eliminates any potential inter-rater reliability. While intra-rater reliability was not formally assessed in this study, all segmentations were checked for accuracy by a second individual (CD).

### Statistical analysis

All analyses were conducted using R (version 4.3.1). MRI-derived data were assessed in relation to polysomnography and obstructive sleep apnea (OSA) endotypes. Summary statistics were calculated for pre-operative data points. Pearson correlation coefficients were calculated between MRI anatomic parameters as well as OSA endotypes and clinical indices. Pre and post-operative comparisons were displayed graphically given the limited number of available data points.

## Results

Our cohort was predominantly female, 45% Hispanic, with a median age of 42 years (Table 1). The median BMI was 40 kg/m^2^ and median AHI was 11/h. MRI as well as endotype parameters are detailed in Table 1. Comparing those with (n = 9) versus those without OSA (defined OSA as AHI≥15/h (n = 13), the median BMI was 39.4 kg/m^2^ vs. 39.2 kg/m^2^ (p = 0.8). The median AHI was 32.2/h versus 5/h. There were no significant endotypic differences, but the hypoxic burden (HBtotal) and event specific ventilatory burden (VB90) significantly differed between groups (Table 1). With respect to MRI morphologic parameters of the upper airway, there were significant differences in retroglossal volume, upper airway volume, and upper airway length, but not tongue volume, tongue fat, or retropalatal volume.

**TABLE 1:** Demographic characteristics of individuals completing baseline demographics (n = 22), upper airway MRI characteristics, as well as polysomnography with endotyping. Parentheses denote mean (SD) and brackets reflect median [IQR].

Demographics and clinical traits by AHI severity			
Variable	AHI ≤15/h	AHI >15/h	p-value
Demographics			
Age	42 [36, 47]	42 [33, 47]	
F	13 (100%)	6 (66.7%)	
M		3 (33.3%)	
Clinical and MRI Parameters			
BMI (kg/m^2^)	39.2 ± 4.5	39.4 ± 2.7	0.9
AHI	5 ± 4.8	32.2 ± 9.4	**<0.01**
HBTotal	8 ± 9	66.5 ± 44.3	**<0.01**
Tongue Volume (mL)	97.8 ± 15.7	106 ± 13.4	0.2
Tongue Fat (%)	13.7 ± 4.2	15.3 ± 5.1	0.5
Retroglossal Volume (mL)	5.9 ± 1.9	9.4 ± 3.1	**0.01**
Upper Airway Volume (mL)	8.9 ± 2.2	13.4 ± 5.5	**0.05**
Upper Airway Length (mm)	57.2 ± 6.6	70.4 ± 4.9	**<0.01**
LG1	0.6 ± 0.2	0.5 ± 0.2	0.25
LGn	0.4 ± 0.1	0.4 ± 0.1	0.56
Chemoreflex Delay (s)	10.4 ± 2.6	10.2 ± 0.9	0.86
ArTH	116.6 ± 26.2	127.4 ± 21.6	0.34
Vpassive	88 ± 23.9	84 ± 11.4	0.64
Vactive	90.7 ± 28.7	86.6 ± 16.5	0.70
Vcomp	2.7 ± 6	2.6 ± 11.7	0.99
Vmin	72.4 ± 19.4	60.8 ± 15.6	0.16
VpassiveT	74 ± 24.6	61.7 ± 12.6	0.189
ArTHresT	132 ± 29.1	148.8 ± 20.2	0.16
VB90	94.7 ± 82.6	561.2 ± 365.4	**<0.01**

Bold value represents the p<0.05.

Within the pre-operative cohort (n = 22), we assessed correlations between MRI-derived upper airway traits and sleep-related physiological parameters. Strong positive correlations (|r| > 0.5) were observed between upper airway volume and hypoxic burden (r = 0.64), upper airway volume and VB90 (r = 0.65), upper airway length and hypoxic burden (r = 0.67), and upper airway length and VB90 (r = 0.71) (Figure 2). Additional correlation plots are provided in the supplement. In particular, tongue fat (%) vs. AHI (Supplementary Figure S1) and airway length vs. AHI (Supplementary Figure S2) are provided.

In the sample of individuals with pre and post-operative MRI of the upper airway as well as pre and post-operative polysomnography (n = 5), descriptive data of clinical, MRI, and sleep parameters are presented in Table 2. Non-parametric comparisons noted significant differences in BMI, Vactive, and Vcomp, but not in MRI parameters, measures of passive anatomy, or loop gain.

**TABLE 2:** Pre vs. Post Analysis: Distributions and non-parametric comparisons of clinical, imaging, and endotype data. Significant relationships (p < 0.05) are bolded.

Variable	Median (pre-op) [Q1, Q3]	Median (post-op) [Q1, Q3]	p-value
Apnea-Hypopnea Index (AHI)	18.1 [2.7, 36.1]	15.2 [0.5, 23.7]	p = 0.7
BMI	**39.3 [38.4, 39.5]**	**28.3 [28.1, 33.4]**	**p < 0.05**
Hypoxic Burden (HBtotal)	49.7 [2.6, 103.8]	12.5 [0.4, 46.5]	p = 0.5
Retroglossal Volume (mL)	8.1 [7.4, 11.6]	4.3 [4.1, 7.9]	p = 0.5
Retropalatal Volume (mL)	3.5 [1.9, 3.5]	2.5 [2.5, 10.3]	p = 0.8
Tongue Fat (%)	15.1 [11.9, 16.0]	9.5 [9.3, 11.7]	p = 0.2
Tongue Volume (mL)	104 [103.2, 104.3]	107 [97.7, 107.1]	p = 1
Upper Airway Length (mm)	72 [55, 73]	67 [55, 67]	p = 0.5
Upper Airway Volume (mL)	11.6 [9.3, 12.9]	6.6 [6.5, 18.2]	p = 0.5
Ventilatory Burden (VB90)	761.3 [451.8, 1,025.5]	333.9 [142.3, 502.8]	p = 0.2
Arthres	128.7 [118.5, 143.5]	132.9 [118.5, 150.5]	p = 1
ArthresT	153.4 [140.6, 164.9]	157.3 [138.9, 169.9]	p = 1
Delay (s)	11.8 [11.2, 11.9]	11.8 [10.5, 12.9]	p = 1
Loop Gain (lg1)	0.6 [0.5, 0.7]	0.7 [0.6, 0.8]	p = 0.9
Loop Gain (lgn)	0.4 [0.4, 0.5]	0.5 [0.4, 0.5]	p = 0.9
VActive	**70.3 [66.7, 74.8]**	**103.7 [102.0, 105.5]**	**p < 0.05**
VComp	**−4.6 [-12.1, 0.5]**	**8.7 [4.7, 14.8]**	**p < 0.05**
VMin	62.7 [52.2, 64.6]	69.9 [61.0, 74.8]	p = 0.3
VPassive	81.3 [72.7, 86.9]	93.6 [88.7, 97.8]	p = 0.2
VPassiveT	56.9 [48.4, 64.3]	75.7 [66.5, 86.7]	p = 0.2

Bold value represents the p<0.05.

## Discussion

Our data highlight the complexity of the relationship between obesity and OSA. Contrary to our expectations, we did not find a robust relationship between BMI and tongue fat % or tongue volume. Likewise, we did not note a correlation between tongue fat % or tongue volume to Vmin. Consistent with prior observations, we did note that airway length correlated strongly with AHI (Segal et al., 2008; K et al., 2011; Lin et al., 2020). Airway length also correlated strongly with HBtotal and VB90. As we and others have noted, the pathogenesis of OSA in obesity is heterogeneous, with age and sex-specific considerations, though our sample was too small to assess sex-related differences in upper airway behavior directly (Nokes et al., 1985; Hang et al., 2025).

With respect to airway length, it is worth noting that our MRI measurements are a reflection of intrinsic airway length and not necessarily of the upper airway under longitudinal tension. The images were averaged over 5 min of tidal breathing so they could be approximated as static volume of functional residual capacity +1/2 tidal volume. This notion is worth highlighting because an intrinsically longer upper airway will tend to be more collapsible than an airway distended by longitudinal tension (Malhotra et al., 2012). This is in relation to tube law, the law of LaPlace, and radius of curvature (Malhotra et al., 2012). Simply, longer tubes not under tension are more collapsible than shorter tubes for the same amount of transmural pressure. Tubes under longitudinal tension, however, have stiffer walls and require greater extrinsic (tissue) pressure to collapse. A simplified form of this tube law is:

$$A=A_{0}+\alpha \;\cdot \text{ Ptm}+\beta \;\cdot \;T$$

Where:

- A_0_ is the baseline (resting) cross-sectional area,
- α is the compliance of the airway wall,
- Ptm is the transmural pressure (Pressure inside–Pressure outside),
- β T represents the stiffening effect from longitudinal tension (T).

As individuals with obesity accumulate visceral fat, there is a loss of tonic and phasic tracheal tug, indirectly influencing the longitudinal stiffening force of the pharynx (Isono, 2012). Additionally, the accumulation of fat within the submandibular space tends to increase external tissue pressure and thereby decrease transmural pressure, lending to airway collapse (Isono, 2012). Thus, pharyngeal propensity for closure can be impacted by obesity-related increases in passive airway length, changes in longitudinal tension, and decreases in transmural pressure (Isono, 2012; Genta et al., 2016).

The relationship between pharyngeal airway length and OSA is well-described, and our findings are consistent with prior data (Pae et al., 1997). Specifically, it has been noted that longer pharyngeal airways portend to increased collapsibility, males tend to have longer upper airways than females relative to body height, and that apneics tend to have longer upper airways in the supine position (Pae et al., 1997; Malhotra et al., 2002).

It bears mention that prior studies have highlighted the importance of tongue and peripharyngeal fat in mediating the pathogenesis of OSA in obesity, with noted improvements in these fat depositions and in turn the AHI with weight loss (Wang et al., 2020). However, there are several key differences between our population and those in the Wang et al., publication. Notably, our population was overwhelmingly female in the peri-menopausal to menopausal age range, compared to 40% men. The disease severity was much milder within our cohort and our overall median pre-operative AHI (11/h) was less than the median AHI of the composite post-weight loss cohort in the Wang et al., study (25/h).

More recently, Xu et al., assessed the upper airway by MRI in an Icelandic population, and demonstrated that obesity influenced upper airway shape, soft tissue volumes, and cephalometric measures, in turn impacting upper airway cross sectional airway at multiple sites (Xu et al., 2025). Notably, Xu et al., found that more obese patients with OSA tended to have larger soft tissue volumes and leaner patients with OSA tended to have a restricted craniofacial features. The also noted increases in anterior-posterior diameter and decreased lateral wall distance with increasing BMI, highlighting that airway shape likely changes with obesity. There are some key differences that should be noted between our populations in considering the heterogeneity with which obesity impacts OSA. In particular, the Xu et al., study population is 81% male, older (mean age 53.7 years), and included a broad range of BMIs, but pre-selected for the presence of OSA (AHI>15 h). We view our findings as complimentary and illustrative of the heterogeneous impact obesity can have on the upper airway.

Notably, there are several limitations to be considered in the current study. Recruitment for the full protocol was low in relation to the COVID-19 pandemic, decreases in bariatric surgery within our center, as well as closure of the MRI facility during the study period. Moreover, the limited number of post-operative datapoints prohibit detailed analysis of concurrent morphologic and endotypic changes and are likely underpowered. We have included a comparison of pre and post-operative MRI and endotype parameters for transparency in Table 2, but realize the limited statistical inference available with such a small number of subjects. Additionally, the median OSA severity for our cohort was mild, the cohort was predominantly women in peri-menopausal or menopausal age range, which may influence fat distribution and OSA endotypes (Wang et al., 2025). Thus, the generalizability of our findings may be limited. Lastly, our findings are predominantly correlational and cannot be viewed as causal given the limitations of the current data set. However, these findings also highlight the variability with respect to how obesity influences upper airway morphology and physiology across different populations. Additionally, they highlight how individual physiology might be incorporated into clinical decision making.

Nonetheless, we view these findings as important in further demonstrating the heterogeneity of obesity, OSA, and the overlap of these two conditions. Further study is needed to determine how anatomy and endotypes evolve with weight loss, whether the mode of weight loss affects this process, and, why OSA might persist following significant weight loss.

## Data Availability

The raw data supporting the conclusions of this article will be made available by the authors, without undue reservation.
