# Supplementary material for: The impact of obesity on upper airway anatomy as assessed by magnetic resonance imaging and obstructive sleep apnea endotypic traits
Source: Front Physiol. 2025 Oct 1;16:1648767. doi: 10.3389/fphys.2025.1648767 (PMC12521235; doi:10.3389/fphys.2025.1648767)
Supplement: Supplementary file 1 [file Presentation1.pptx]

## Slide 1
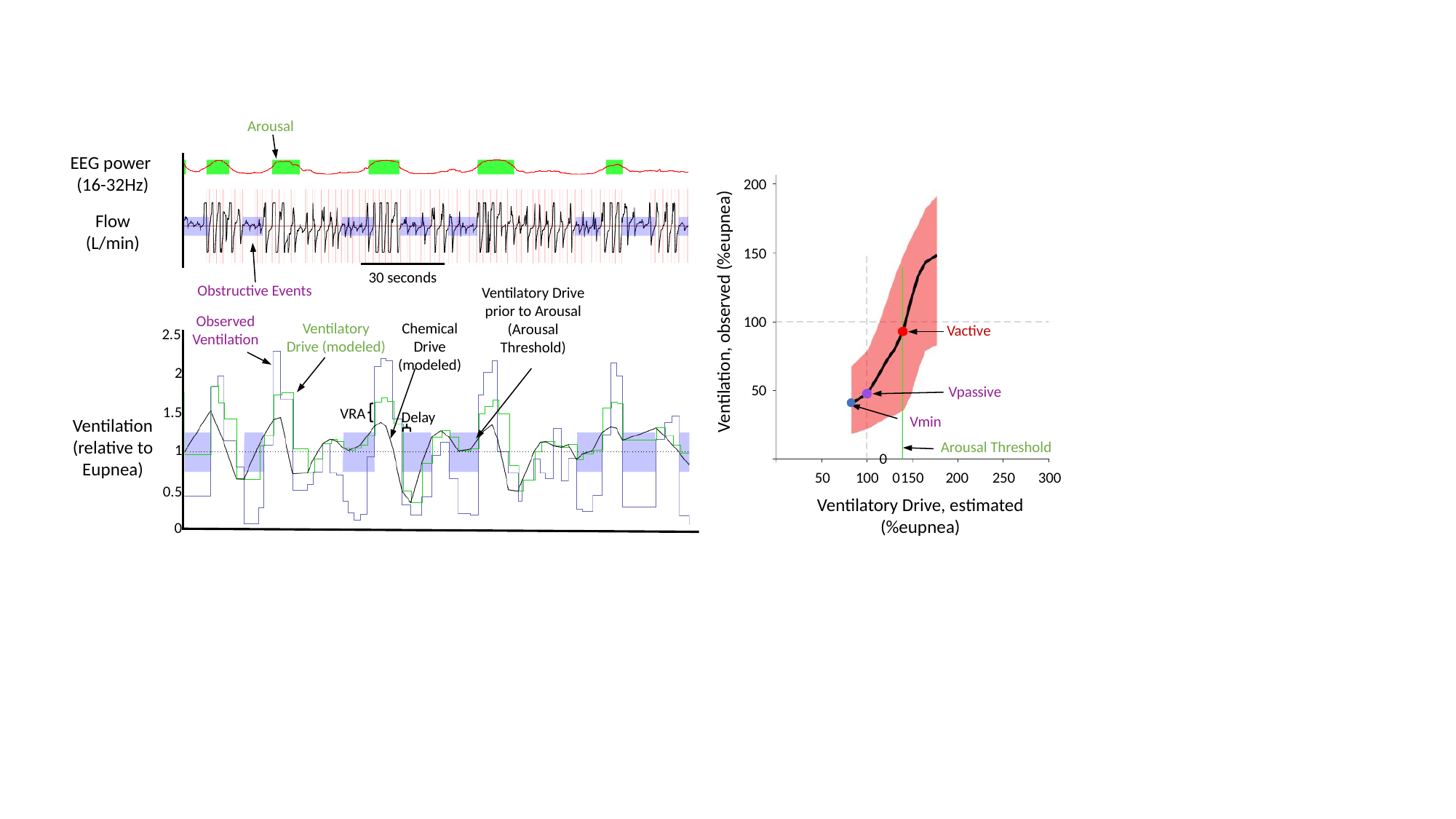

Arousal
EEG power
(16-32Hz)
Flow
(L/min)
30 seconds
Obstructive Events
Ventilatory Drive prior to Arousal (Arousal Threshold)
Observed Ventilation
Chemical
Drive
(modeled)
Ventilatory
Drive (modeled)
2.5
2
1.5
VRA
Delay
Ventilation (relative to Eupnea)
1
0.5
0
200
150
Ventilation, observed (%eupnea)
100
Vactive
50
Vpassive
Vmin
Arousal Threshold
0
50
100
0
150
200
250
300
Ventilatory Drive, estimated (%eupnea)
