# Supplementary material for: The impact of obesity on upper airway anatomy as assessed by magnetic resonance imaging and obstructive sleep apnea endotypic traits
Source: Front Physiol. 2025 Oct 1;16:1648767. doi: 10.3389/fphys.2025.1648767 (PMC12521235; doi:10.3389/fphys.2025.1648767)
Supplement: Supplementary file 2 [file DataSheet1.pdf]

Supplemental data

Variable	Description	Unit
**LG1**	"Loop gain" is the magnitude of reflex ventilatory drive response to a change in ventilation; it describes the ventilatory control sensitivity without chemoreflex delay effects.	NA
**LGn**	"Overall instability" is the magnitude of reflex (opposing) ventilatory drive response to a change in ventilation; it describes the ventilatory control stability and includes the effect of circulatory delays.	NA
**Delay**	Chemoreflex delay, i.e. the latency between a drop in ventilation and a subsequent reflex rise in ventilatory drive.	s
**VRA**	Ventilatory response to arousal – the increase in ventilatory drive that is attributable to arousal/wakefulness.	%
**ArThres**	"Arousal threshold" is the level of ventilatory drive that causes arousal from sleep, in percentage of eupneic levels.	%
**Vpassive**	"Collapsibility" is taken as the level of ventilation at eupneic ventilatory drive, i.e. collapsibility under passive conditions. Low values indicate greater collapsibility.	%
**Vactive**	The level of ventilation at elevated ventilatory drive, i.e. collapsibility under active conditions. Low values indicate greater active collapsibility.	%
**Vcomp**	Equal to Vactive minus Vpassive. Reflects the increase in airflow due to activation of the pharyngeal dilator muscles.	%
**Vmin**	"Collapsibility" is taken as the level of ventilation at minimal ventilatory drive, i.e. collapsibility under hypotonic conditions. Low values indicate greater collapsibility.	%
**VpassiveT**	Transformed Vpassive; Constraint: x>100 is set to 100; Square-root transformation per 1-(1-x)0.5.	%
**ArThresT**	Transformed ArThres; Constraint: x<1 is set to 1 (here, 1 indicates 100%). Transformation used = 1+(x-1)0.5.	%
**VpassiveTA**	Alternate transformation of Vpassive (Constraint: x>99.5 is set to 99.5, Transformation: α[1+(x-1)0.33], rescaling factor α=1.2046)	%
**VactiveTA**	Alternate transformation of Vactive (Constraint: x>99.5 is set to 99.5; Transformation: α[1+(x-1)0.33], rescaling factor α=1.2046)	%
**VB90**	Event specific area under "ventilation" curve, using data from breaths within scored events that are 90% or less of eupnea (%eupnea x time / hour of sleep).	%

Supplemental Table 1: Definitions and calculations for obstructive sleep apnea endotypic traits as listed in the PUPbeta data dictionary. Ventilatory burden is as described by Staykov et al.

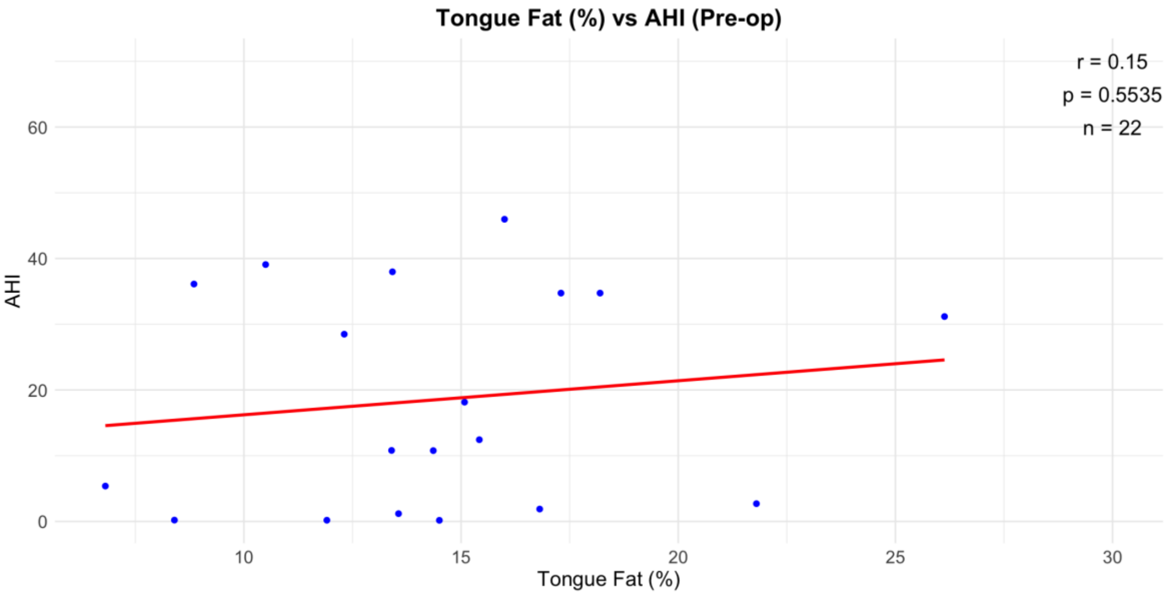

Supplemental figure 1: Tongue fat (%) versus apnea hypopnea index (AHI) within the pre-operative cohort. Correlation coefficient (r=0.15, p=0.55, n=22).

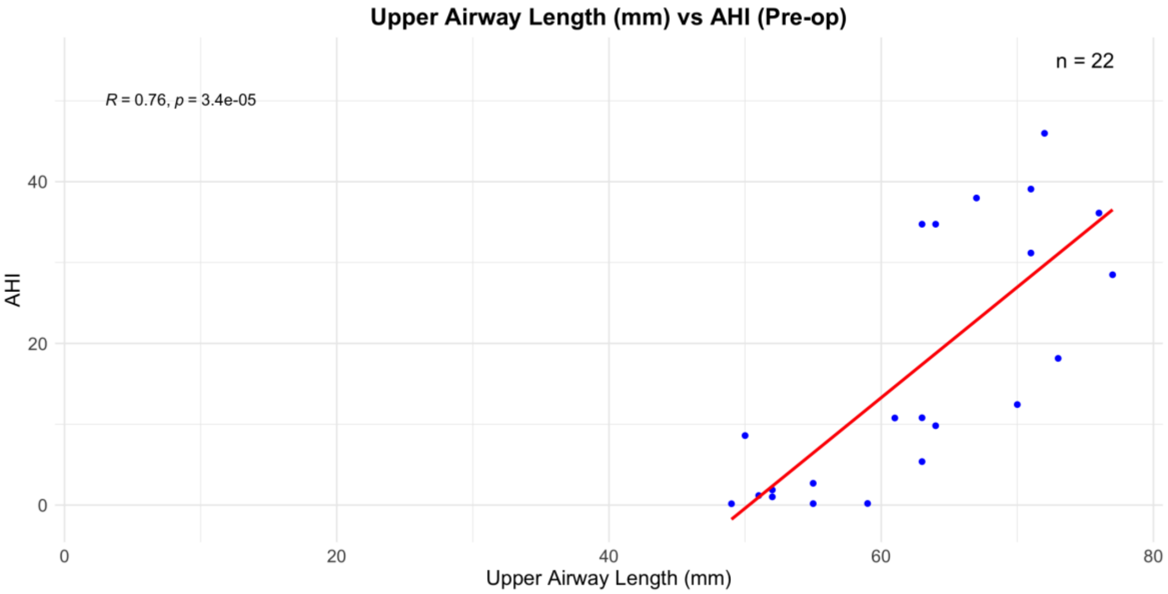

Supplemental figure 2: Airway length (mm) versus apnea hypopnea index (AHI) within the pre-operative cohort. Correlation coefficient (r=0.76), p<0.001, n=22.
